# Optimizing Multicomponent Interventions to Improve Child Mental Health Following Reintegration From Institutions: Protocol for a Factorial Randomized Trial

**DOI:** 10.2196/93033

**Published:** 2026-07-16

**Authors:** Leyla Ismayilova, Narmin Guliyeva, Fuad Ismayilov, Emma Heidorn, Fred Ssewamala, Kerim Munir, Deborah Gorman-Smith, Parvin Muslumzada, Emily Claypool, Loren Beard, Donald Hedeker

**Affiliations:** 1 Crown Family School of Social Work, Policy, and Practice, University of Chicago, 969 E 60th St, Chicago, IL, 60637, United States, 1 773 702-1250; 2 Department of Psychiatry, Azerbaijan Medical University, Baku, Azerbaijan; 3 National Mental Health Center, Baku, Azerbaijan; 4 Silver School of Social Work, New York University, New York, NY, United States; 5 Harvard Medical School, Harvard University, Boston, MA, United States; 6 Division of Developmental Medicine, Boston Children's Hospital, Boston, MA, United States; 7 Department of Family Medicine and Community Health, University of Wisconsin–Madison, Madison, WI, United States; 8 Department of Sociology, Faculty of Arts and Sciences, Harvard University, Boston, MA, United States; 9 Department of Public Health Sciences, Biological Sciences Division, The University of Chicago, Chicago, IL, United States

**Keywords:** mental health, children, deinstitutionalization, family strengthening, economic empowerment, Eurasia

## Abstract

**Background:**

The legacy of the Soviet childcare system has contributed to persistently high rates of child institutionalization across many countries in Eastern Europe, the Caucasus and Central Asia. Although countries throughout the region have adopted deinstitutionalization and family reunification policies, most programs provide limited support to address children’s mental health needs or family functioning during the critical transition from institutional care to family and community reintegration.

**Objective:**

This study compares and optimizes 3 prevention and early intervention approaches to improve mental health outcomes for children with a history of institutional care. Using the multiphase optimization strategy, the study experimentally evaluates which interventions—family strengthening, trauma-focused child mental health services, or economic empowerment—yield meaningful improvements in child mental health.

**Methods:**

This randomized clinical trial used a full 2³ (2×2×2) factorial design. A total of 439 children aged 7 to 12 years with a history of institutional placement and subsequent family reunification, along with 305 primary caregivers (parents or kin caregivers), were recruited from the 3 largest cities in Azerbaijan. Participants were randomly assigned to 1 of 8 experimental conditions, each receiving different combinations of the 3 interventions plus usual care. The family strengthening intervention consisted of a 12-session multifamily group program designed to improve parent-child relationships and supportive parenting. The asset-based economic empowerment intervention included opening a Matched Child Development Account with a US $50 seed deposit, 2:1 matched savings up to US $20 per month, and an 8-week financial education program for caregivers and children. Data on child mental health outcomes (self-esteem, depression, posttraumatic stress disorder, attention-deficit/hyperactivity disorder, internalizing and externalizing problems), cognitive and social processes (executive functioning, working memory), social and academic functioning (eg, grades), family functioning, and economic well-being were collected at baseline, 1-year, and 2-year follow-ups using multi-informant surveys (child, caregiver, and teacher reports), digital cognitive tasks (Wechsler Intelligence Scale for Children, Fifth Edition and Cambridge Neuropsychological Test Automated Battery), and clinical assessments.

**Results:**

Recruitment occurred between 2022 and 2023, with 2-year follow-up data collection completed in 2025. Intervention effects analyses are ongoing and are expected to be completed in 2026.

**Conclusions:**

This study will identify the most effective and efficient combination of interventions to support the mental health of children reintegrating from institutional care. The findings are expected to provide important evidence to support deinstitutionalization and family reunification initiatives in the region and strengthen the global evidence base on effective, scalable community-based interventions addressing multilevel determinants of mental health among at-risk children in low- and middle-income countries.

## Introduction

The region of Eastern Europe and Central Asia has the second-highest rate of children living in institutional care worldwide: 232 per 100,000 compared to a global average of 105 per 100,000 [1]. Due to the economic crisis following the collapse of the Soviet Union, many countries in the region hosted a population of “social orphans,” children who have at least one living parent but were voluntarily placed in institutions due to socioeconomic reasons (eg, poverty, divorce, labor migration) [2]. The experience of institutionalization can put children at high risk for social, emotional, and behavioral difficulties due to social isolation, deprivation, a high child-to-service provider ratio [3-5], and the trauma of separation from their family [6,7].

Acknowledging the detrimental effects of institutionalization, Azerbaijan and many other countries in the region have implemented national deinstitutionalization programs aimed at closing institutions and reunifying children with their families of origin [6,8]. These programs typically provide case management but do not offer support during reunification to address children’s mental health needs, family functioning, or parental distress [9]. Furthermore, most deinstitutionalization programs do not address family-level poverty, which often spurred initial institutional placement, creating a risk of poor psychosocial outcomes associated with economic precarity.

Prevention and early intervention reduce children’s risk of developing mental health disorders in adulthood, improve integration into society, and concurrently impact a number of psychosocial outcomes [10-12]. Yet, there are no evidence-based, culturally tailored, preventive mental health interventions available for institutionalized children in Azerbaijan and other countries in the region. Existing interventions, primarily formulated in high-resource countries, such as the United States, require highly skilled clinicians and are not adapted for developing countries with limited financial and mental health resources [13,14]. Using innovative research methodologies to develop early prevention programs is essential to optimize the well-being of reunifying children and families in low- and middle-income countries (LMICs) such as Azerbaijan.

This paper describes a study protocol for a US National Institute of Child Health and Human Development/NICHD (R01-HD099847) study titled “Optimizing prevention approaches for children reintegrating from orphanages in Azerbaijan.” The study is registered with the ClinicalTrials.gov registry (NCT05396625). Trial registration was completed on May 19, 2022, and the recruitment start date was May 1, 2022; therefore, the trial was registered shortly after recruitment had begun and was not prospectively registered.

Although recruitment and data collection have been completed, this protocol paper documents the study design, intervention components, measurement approach, ethical safeguards, and prespecified analytic plan prior to the publication of the main trial findings. No intervention effects or primary outcome results have yet been published. Baseline characteristics are reported solely to describe the enrolled sample and assess comparability across randomized conditions, not as evidence of intervention effectiveness.

## Methods

### Study Design

This randomized clinical trial used a full 2³ (2×2×2) factorial design based on the multiphase optimization strategy (MOST) framework to test and compare three intervention approaches: (1) a family-strengthening intervention, (2) trauma-focused child mental health services, and (3) asset-based economic empowerment via Child Development Accounts (CDAs).

Multicomponent interventions are often tested as “bundles,” making it difficult to disentangle the contributions of individual components. The MOST is a systematic method that allows for the testing of the independent effects of different intervention components to identify their most optimal combination [15]. While multiarm randomized controlled trials are costly, often limited in arms, and require larger sample sizes, the MOST uses a factorial design. By comparing combinations of experimental conditions, the study can have a greater number of conditions without a substantial increase in sample size. This is advantageous in LMICs, where it is essential to implement only the most efficacious aspects of a program. The MOST consists of three phases: (1) preparation, (2) optimization, and (3) evaluation of the optimized intervention in a randomized controlled trial [16]. This study falls within the optimization phase.

This study includes 3 factors—the intervention approaches—each with 2 levels (delivered/“on” or not delivered/“off”). This is a full 2×2×2 factorial design, meaning that the study has 8 different experimental conditions or groups (2^3^=8) [17]. Child-parent dyads (n=439) were randomly assigned to 1 of 8 groups (about 54 dyads per group), which determined the combination of interventions they received. Given this design, half of the sample (n≈220) received each intervention (eg, families assigned to groups 1‐4 received the family-strengthening intervention and were compared with families from groups 5‐8; Table 1).

**Table 1.** Experimental conditions^a^.

Group	Usual care	Experimental interventions		
	Standard deinstitutionalization program	Family strengthening	Trauma-focused mental health services	Economic empowerment
1	On	On	On	On
2	On	On	On	Off
3	On	On	Off	On
4	On	On	Off	Off
5	On	Off	On	On
6	On	Off	On	Off
7	On	Off	Off	On
8	On	Off	Off	Off

a Child-parent dyads (n=439) are randomly assigned to 1 of 8 experimental conditions (~54 dyads per group), determining how many and which intervention components participants receive.

### Objectives

This trial has three study aims:

1. To refine, test, and compare the effects of 3 interventions (family-strengthening intervention, mental health services, and economic intervention) on mental health outcomes (eg, posttraumatic symptoms, depression, anxiety, internalizing and externalizing problems) and cognitive functioning (eg, working memory, executive functioning, impulsivity) among 7‐ to 12-year-old children with a history of institutional care in Azerbaijan.
2. To examine the role of hypothesized intervention mediators (eg, emotion regulation, supportive parenting, parental stress) and moderators (eg, child’s and caregiver’s age, gender, length of time in institution, family structure, intervention adherence) in improving child mental health outcomes.
3. To explore the facilitating factors and barriers to the implementation and participation in each intervention component using qualitative interviews with participants and service providers.

The study also aims to conduct a secondary analysis to estimate the preliminary costs (operations and personnel) of each intervention component relative to usual care alone.

### Sample and Eligibility Criteria

The sample consists of 439 caregiver-child dyads living in 3 cities in Azerbaijan: Baku, Sumgait, and Ganja. Caregivers and children must meet the eligibility criteria to participate and commit to study participation to be enrolled. Eligible children (1) are 7 to 12 years old at baseline, (2) have a history of institutionalization (for at least 1 month), and (3) have been reunited with a caregiver (biological parent or kin relative) within a year before the start of the study.

Eligible caregivers (1) are a biological parent or kin relative (eg, grandparent) who serves as the primary caregiver;, (2) reunified with the child before the study, and (3) are at least 18 years old at baseline. Children or caregivers with significant cognitive, behavioral, and/or mental health impairment (eg, severe developmental disorder) that may interfere with their ability to consent to, benefit from, or participate safely in the program are excluded from participation. More than 1 eligible child in a family may be enrolled in the study. Enrollment requires consent from the caregiver and assent from the child (see Multimedia Appendix 1 for sample consent and assent forms).

### Power Calculations

Power calculations were conducted using the G*Power software [18,19]. To account for the repeated-measures design, power estimates were modeled across a range of effect sizes at 2 assumed autocorrelation values (ρ). Analyses were performed for a 2-tailed test with a type 1 error rate of 0.05 and desired power of 0.80, assuming a final analyzed sample size of 360 child participants (180 participants per intervention type) after a projected 10% attrition from an initial planned sample of 400 child participants (200 participants per intervention type). The results indicate that for continuous outcomes, 80% power is achieved for effect sizes between 0.09 and 0.12. The study is sufficiently powered to detect small-to-medium effects [20] for continuous outcomes over the entire follow-up period. In the MOST design, the study is powered based on the intervention component with the smallest effect size—in this case, the family-strengthening component.

### Preliminary Research

The intervention components were chosen based on the findings from a qualitative study [7] conducted in Azerbaijan from 2014 to 2016 with children aged 8 to 16 years who had a history of institutionalization and their caregivers (n=47). In-depth interviews were conducted with children, caregivers, and experts working with these families. Three primary areas for intervention were identified: addressing children’s mental health needs, strengthening family relationships, and tackling financial challenges during reunification [21,22].

A pilot feasibility study was carried out from 2017 to 2019 to adapt and pretest the family-strengthening intervention with 20 reunified families. The adapted intervention was pilot-tested in partnership with 2 local organizations: SOS Children’s Villages-Azerbaijan (community-based service provider) and the National Mental Health Centre (NMHC; research and delivery of mental health services). This study uses an adapted version of SAFE Children, a family-based preventive intervention originally developed and tested in the United States for at-risk families in low-income urban neighborhoods [23]. SAFE Children was selected because of its strong strengths-based approach and its demonstrated acceptability to families of varying sizes and compositions—a critical feature when working with families affected by institutional care, many of whom do not live in traditional 2-parent households. The research team adapted SAFE Children to the cultural context of Azerbaijan and trained 10 local facilitators (eg, social workers, family support workers) to deliver the adapted version, named “Bütöv Ailə” (Families Together in Azerbaijani). The program consists of 12 weekly multifamily group sessions focused on improving parent-child relationships, supportive parenting practices, communication skills, and child behavior management, with additional content addressing unique challenges faced by families during reunification after institutional care (eg, stigma, fear of abandonment).

### Theoretical Framework

The study is informed by theories of family resilience [24,25] and emotional regulation [26,27]. Developmental theory suggests that adversity and trauma experienced in early childhood may interfere with typical developmental trajectories, which can result in emotional and behavioral problems [28,29]. Yet, with appropriate resources and support, children may develop resiliency and overcome disadvantages [30,31]. In addition, asset theory [32] posits that assets—such as savings, homeownership, education (human capital), stable housing, and small business ownership—unlike cash benefits, which can be quickly depleted, provide significant long-term psychological benefits beyond economic outcomes, such as reducing financial stress and enhancing aspirations and hopes for the future [33,34].

Therefore, it is hypothesized that strengthening children’s individual capacities and external resources can balance the effects of adversity and improve mental health outcomes. Enhancing children’s coping skills, strengthening child-parent relationships, reducing parental stress, and improving family economic well-being by incentivizing savings and building assets will result in children demonstrating fewer symptoms of (1) internalizing problems (depressive or anxious mood), (2) externalizing problems (aggressive, delinquent, or disruptive behaviors), and (3) posttraumatic stress (Textbox 1).

Textbox 1. **Conceptual model.**

**Intervention components**

- Trauma-focused mental health services (psychoeducation, attunement, coping, and self-regulation skills)
- Family strengthening intervention (multifamily groups on family communication, conflict resolution, and family support)
- Asset-based economic intervention (Matched Child Savings Accounts, financial education, and mentoring)

**Proximal mediators**

- Emotion regulation (decreased parental distress, impulsivity)
- Positive family relations (supportive parenting, quality of child-parent and family relationship)
- Economic well-being (food security, household assets, housing, employment)

**Child mental health outcomes**

- Constructs
  - Cognitive and executive functioning (attention, cognitive control or inhibition, working memory)
  - Social processes (emotion recognition, affiliation, and attachment)
- Clinical outcomes
  - internalizing problems (depressive, anxiety, trauma symptoms)
  - externalizing problems (aggressive, antisocial, or delinquent behaviors)

- Functional outcomes (academic performance, peer relations)

### Intervention A: Family Strengthening

This study uses an adapted version of the family-strengthening intervention “SAFE Children” to improve child-parent relationships, enhance supportive parenting strategies, and prevent emotional and behavioral problems among at-risk children [23]. SAFE Children was initially designed for at-risk families from low-income US neighborhoods and uses a multi-family group approach to build social support. Including multiple families is an efficient service delivery model, fostering support among participants and improving parent-child interactions directly [35].

Families participated in 12 weekly sessions, with themes including identifying family strengths, developmental expectations, communication skills, behavior management, parental distress management, and conflict resolution. The adapted intervention incorporates topics that address unique issues faced by families during reunification (eg, attachment issues, children’s feelings of resentment, parental guilt, and shame). In response to the findings of the pilot study, sessions were offered on weekends to accommodate families’ schedules. The intervention developer and coinvestigator (DB-S) oversaw the training and intervention adaptation process to ensure fidelity to its core components. The intervention is delivered in multifamily groups of 5 families. Children and caregivers attend sessions together, separating into child- and caregiver-specific activities only when required by the curriculum. To strengthen family engagement and reinforce skills at home, all household members involved in child-rearing are encouraged to participate, including siblings who do not meet study eligibility criteria.

### Intervention B: Mental Health Services

Families were connected to clinicians from the NMHC to receive child mental health services. Services included a clinical assessment performed by a psychiatrist, the development of an individual care plan, and assignment to a mental health clinician—typically a child psychologist—who provided services to the child and caregiver. The trauma-focused child mental health services were grounded in the Attachment, Regulation, and Competency framework [36]. All clinicians delivering this intervention received additional training in trauma-focused mental health approaches, including Attachment, Regulation, and Competency, and participated in biweekly group supervision delivered by a bilingual licensed psychologist from the United States. The clinicians delivering this intervention were not involved in conducting research activities. As a public entity, the NMHC provides all mental health services to the public free of charge. Children typically attended individual sessions once a week (30‐45 min), with parent or caregiver involvement incorporated as clinically indicated. Up to 12 sessions were offered; however, services could be extended based on individual treatment plans and clinical needs.

### Intervention C: Asset-Based Economic Empowerment

#### Overview

The economic intervention involves 3 components: CDAs, financial education sessions, and monthly mentoring. Under Azerbaijan’s National Social Targeted Assistance Program, low-income families are eligible for small monthly cash allowances. While cash benefits provide immediate relief, they may not fully lift families out of poverty [37,38]. In contrast, CDAs encourage savings and asset investment (eg, a child’s education, housing, or small business development), aiming to prevent economic shocks, reduce poverty-related parental stress, and increase family stability [39,40].

#### Child Development Accounts

Each child received a US $50 seed deposit to open a dedicated savings bank account, and caregivers were encouraged to make small monthly contributions. Savings of up to US $20 per month (“matching cap”) were matched by project funds at a 2:1 ratio—meaning that for every US $1 saved by the family, the project contributed US $2—and deposited into a separate CDA account managed by the study. These matched savings could only be used toward 3 critical investments essential for family stability and well-being: the child’s education (eg, school expenses and tutoring fees), starting or expanding a small business (eg, a nail salon, animal husbandry, or baking business), or housing improvements. If a family consistently saves US $20 each month for the full 24 months and makes no withdrawals, the total accumulated amount per child would reach US $1490 at the end of the 2-year study. This comprises the US $50 seed deposit, US $480 in family contributions (US $20×24 months), and US $960 in matched funds (US $40×24 months at the 2:1 matching rate).

In Azerbaijan, this amount could cover approximately 1 year of tuition at a private university or a substantial portion of living expenses for a student attending a public university. For younger children, it could fund 1 to 2 years of supplemental educational support, such as tutoring or academic enrichment programs, which are often necessary to prepare for competitive national university entrance examinations. For low-income families, particularly those raising multiple children, accumulating this level of savings is often unattainable without external support.

#### Financial Education

Families participated in 8 weekly 1-hour financial education sessions delivered in groups of 5 families. During each session, caregivers and children attended separate age-appropriate group sessions conducted simultaneously in different rooms. Caregivers’ sessions covered topics such as the importance of saving, sources of income, income generation, family budgeting, and resource management. The curriculum was originally developed by FS, a coinvestigator, and has been implemented with children and families in Sub-Saharan Africa participating in asset-based economic empowerment interventions. Based on the Junior Achievement *More Than Money* program, children participated in 8 weekly interactive, game-based sessions that introduced financial literacy and entrepreneurship concepts in an engaging and age-appropriate format. Through hands-on activities, role-playing, and educational games, children learned practical skills in earning, spending, saving, and money management. The sessions aimed to develop the ability to make informed financial decisions, understand trade-offs associated with spending and saving choices, and explore basic entrepreneurship concepts while identifying how their interests, strengths, and talents can be developed into future educational and career opportunities. Adapted for the Azerbaijani cultural context, the curriculum promotes both financial capability and positive future orientation. Both curricula were tailored to the cultural and economic context of low-income families in Azerbaijan. After completing financial education, families met monthly with a mentor to review their savings progress.

### Usual Care

In addition to the 3 experimental interventions, all participants were eligible to receive standard public services under Azerbaijan’s national deinstitutionalization program for family reunification and reintegration. This included case management, referrals to public schools, and social support services during the transition and reintegration process—such as medical-social services, sociopedagogical support (education and development), and linkage to the general social protection system (financial and material assistance for vulnerable families). The government’s standard support focuses on case management, access to social and medical-psychological services through state institutions, and basic follow-up after reunification. The emphasis is on returning children to a family environment and connecting them to existing public services.

### Intervention Quality Assurance and Implementation Measures

For each component, we will analyze data regarding participation (eligibility, enrollment, and attrition rates), intervention fidelity (via facilitator and participant rating forms completed at the end of each session), safety (number of adverse events), intervention acceptability (participant satisfaction, session attendance, and associations between satisfaction and attendance), perceived usefulness (relevance and utility), and cross-arm contamination.

Two interim analyses will be conducted when 33.3% and 66.7% of the planned sample size have completed 6-month follow-up assessments. With respect to efficacy, if any interim analysis shows statistical significance (*P*<.05) for all primary outcomes, the study will be stopped, indicating a significant benefit of 1 intervention over another. With respect to futility, if the effect size estimates suggest a null result is likely given the planned total sample size, the study may be stopped to conserve resources. A post hoc power analysis will assess the minimum sample size needed to achieve 80% power *and* reject the null hypothesis at each interim analysis. If this exceeds the planned 400 dyads for self-reported outcomes, the investigative team will discuss whether to stop the study for futility or modify the design to avoid it, such as increasing enrollment or hiring additional staff.

### Participant Timeline

Families were recruited in 3 major cities (Baku, Sumgayit, and Ganja). Trained local research assistants (RAs) distributed recruitment materials to recruiters who invited families to join the study. Local research staff provided additional information to interested candidates and obtained informed consent. To minimize potential pressure on children and parents, consent was obtained by a research staff member rather than by representatives of local authorities.

After obtaining informed consent, RAs conducted a screening interview to determine participant eligibility. Potential participants were offered a choice of either US $1 or equivalent nonmonetary compensation (eg, toys, football) for completing the interview. To promote retention, participants provided their addresses, email addresses, and telephone numbers at baseline, along with contact information for at least 3 other individuals who could assist in reaching them. After completing the baseline survey, families were informed of their randomized intervention assignment.

### Randomization

The principal investigator (PI) conducted the randomization prior to the start of recruitment. Eight computer-generated random numbers (ranging from 0 to 100) were assigned to the 8 intervention conditions and ranked from lowest to highest to determine the allocation sequence within each city. The first 5 families recruited in a city were assigned to the first intervention condition in the sequence. After 5 families had been assigned to each intervention condition, allocation resumed at the beginning of the sequence. To reduce predictability, the random numbers were generated from a range of 0 to 100 rather than 1 to 8. A secure, web-based, password-protected database was used to maintain the randomization record.

Data collectors were masked to participants’ intervention group assignments. To foster fidelity to the intervention manuals and maintain the integrity of each separate intervention, facilitators delivered only 1 type of intervention and were not trained in the specific content of the other intervention strategy. Due to the nature of the recruitment procedures, participants could not be blinded to the existence of other intervention conditions.

### Outcome Measures

As presented in Table 2, the primary mental health outcomes include child-reported symptoms of depression (20-item Center for Epidemiological Studies Depression Scale for Children) [41], posttraumatic stress (8-item Children’s Revised Impact of Events Scale) [42], self-esteem (20-item Tennessee Self-Concept Scale-Second Edition) [43], attention-deficit/hyperactivity disorder (ADHD) symptoms (caregiver and teacher-reported 19-item ADHD Rating Scale-IV) [44], and general social-emotional functioning (caregiver and teacher-reported 25-item Strengths and Difficulties Questionnaire) [45]. Grades from school records and teacher-reported school behaviors (eg, completing homework, fights with other students) are also outcomes of interest. The Strengths and Difficulties Questionnaire has been validated in the Azerbaijani language [46]. Child mental health outcomes among families randomized to the mental health services were also assessed by a clinician-administered Health of the Nation Outcome Scales for Children and Adolescents (15 items) [47].

**Table 2.** Measurement instruments^a^.

Outcomes and measurement instrument	Respondent
Outcomes (study aim 1)	
Internalizing and externalizing problems	
Center for Epidemiological Studies Depression Scale for Children	Child
Strengths and Difficulties Questionnaire	Caregiver, teacher
ADHD Rating Scale IV	Caregiver, teacher
Health of the Nation Outcome Scales Child and Adolescent Mental Health	Clinician rated
Traumatic symptoms	
8-item Children’s Revised Impact of Events Scale	Child
Cognitive and executive functioning (attention, cognitive control-response inhibition or impulsivity, working memory)	
*The Cambridge Neuropsychological Test Automated Battery*, a digital battery of nonverbal cognitive tasks to assess motor function, processing speed, working memory, executive functioning, and emotion recognition bias through the Motor Screening Task, Reaction Time, Spatial Span, Stockings of Cambridge, and Emotion Bias Task, respectively. *The Wechsler Intelligence Scale for Children* Block Design and Picture Span subtests to assess nonverbal reasoning and visuospatial working memory, respectively.	Child (Digital paradigm tasks)
Self-concept	
Tennessee Self-Concept Scale-Second Edition	Child
Social support	
Social Support Scale for Children	Child
School performance	
Academic performance (grades), school behaviors	Teacher, admin records (school)
Child-parent mediators (study aim 2)	
Child-parent relationship	
Brief Family Relationship Scale; Multidimensional Scale of Perceived Social Support	Child, caregiver
Parenting skills and practices	
Parenting Styles and Dimensions Questionnaire; Child Discipline	Caregiver, child
Parental distress	
21-item Depression Anxiety Stress Scale; Brief COPE	Caregiver
Socioeconomic deprivation, savings, and assets	
The rate of savings, asset accumulation, employment, type and stability of housing, financial security, and attitudes to saving and income-generating activities	Caregiver, admin records (bank statements)
Moderators	
Child’s sociodemographics	
Sociodemographic questionnaire (child’s age, gender, birth order)	Child, caregiver
Parent and family characteristics	
Sociodemographic questionnaire (parent’s gender, age, marital status, employment status, family size, family structure)	Caregiver
Parent	
Adverse Childhood Experiences International Questionnaire	Caregiver
Service use	
Types of services, the number of sessions attended	Admin records

a This table outlines outcomes, mediator, and moderators of interest.

Additional child-reported measures include social support (24-item Social Support Scale for Children) [48] and parental discipline (11-item Child Discipline Index) [49]. Child cognitive functioning was assessed using selected measures from the Wechsler Intelligence Scale for Children, Fifth Edition (WISC-V) [50] and the Cambridge Neuropsychological Test Automated Battery (CANTAB) [51]. WISC-V measures included Block Design, which assesses visuospatial reasoning and problem-solving abilities, and Picture Span, which evaluates visual working memory. CANTAB measures included the Emotional Bias Task, which assesses emotional processing and interpretation bias; the Motor Screening Task, which evaluates sensorimotor function and task comprehension; Reaction Time, which measures processing speed and attention; the Stockings of Cambridge, which assesses executive functioning and planning; and Spatial Span, which measures visuospatial working memory capacity.

Caregiver-reported measures include parenting style (32-item Parenting Styles and Dimensions Questionnaire, Short version) [52]; parental mental well-being symptoms, including depression, anxiety, and stress (21-item Depression Anxiety Stress Scale) [53]; parental experiences of childhood trauma (19-item Adverse Childhood Experiences International Questionnaire) [54]; family relationships (19-item Brief Family Relationship Scale) [55], coping strategies (28-item Brief COPE) [56]; and social support (12-item Multidimensional Scale of Perceived Social Support) [57]. The caregiver survey also assessed economic well-being (eg, employment status, financial security) and saving attitudes.

The baseline questionnaire collected information on sociodemographic characteristics (eg, child and caregiver age and gender), family composition (eg, caregiver marital status and household size), and the child’s institutional history (eg, age at placement and type of institution). For participants receiving the economic intervention, bank statements provided data about the monthly savings and asset accumulation rate.

### Data Collection

Children, their caregivers, and their teachers completed surveys at baseline, year-1, and year-2 follow-up. Child and caregiver data were collected through interviewer-administered surveys conducted separately in Azerbaijani by trained graduate-level psychology students serving as RAs. Assessments were conducted at local field offices using iPads and the cloud-based Qualtrics survey platform and required approximately 50 to 60 minutes to complete. Teacher surveys were completed independently. All surveys contained the same scales, except for demographic data that do not vary over time (eg, ethnicity, age). Except for nonverbal digital cognitive tasks (WISC and CANTAB), all instruments were translated into Azerbaijani and tested in a pilot feasibility study. The CANTAB battery consists of simple computer-administered nonlinguistic tasks considered highly precise and largely independent of cultural and language differences [58].

Intervention participation and utilization data (eg, attendance, session completion, and types of services received) were collected from intervention administrative records. Information about children’s academic performance (eg, grades) was collected from school records.

### Postintervention Qualitative Interviews

Semistructured interviews will be conducted with children, caregivers, and service providers (n=60) representing each intervention condition following the completion of intervention sessions and follow-up assessments. Interviews will explore participants’ experiences with the interventions, including perceived benefits, acceptability of intervention components, impacts on family relationships and child well-being, and barriers and facilitators to participation. Service providers (eg, mental health clinicians, family strengthening facilitators, and financial education coaches) will provide additional perspectives on implementation, training, organizational, and cultural factors affecting intervention delivery. Participants will be randomly selected from the 7 active intervention conditions within each city; the control condition will be excluded from qualitative sampling.

### Analytical Strategy

Descriptive statistics will characterize the sample and assess the variables’ psychometric and distributional properties (eg, potential outliers). The analysis will also include process measures for intervention implementation and cost estimation of intervention delivery, following Weinstein et al [59] and Gold’s [60] guidelines. Additional analyses will be performed in conjunction with the study aims:

- Aim 1: To estimate the effects of intervention components on child mental health outcomes, differences in outcomes (eg, depression score) will be tested across 3 time points (baseline, 1 year, and 2 years), under an intent-to-treat basis, using linear mixed models. This approach accounts for the autocorrelation of observations nested within individuals over time. We will use logistic functions for binary outcomes (eg, mental health symptoms above the clinical cutoff), Poisson or negative binomial functions for count outcomes (eg, the number of aggressive behaviors), and linear functions for continuous variables (eg, self-esteem score). Statistical inference will be based on F-statistics and their associated *P* values for between-subject effects (the intervention assignment × time terms). To estimate the main effects for the 3 intervention components, we will determine if there is a difference in change across time using the baseline as the reference point (eg, 1 year vs baseline; 2 years vs baseline). We will also include 2-way interactions between components (eg, Family Strengthening by Economic Empowerment by Time interaction). Since we will use effect (–1, 1) coding rather than dummy (0, 1) coding, the power for tests of main effects and interactions will be identical for similarly sized effects. Covariates will include baseline attributes for outcome variables (eg, child’s age and gender, caregiver characteristics).
- Aim 2: To test which intervention component is mediated by hypothesized mediators, including (1) supportive parenting (eg, quality of child-parent relationships), (2) emotion regulation (eg, self-regulation), and (3) parental stress (eg, stress response). We will use a 2-step process [61] to test (1) a measurement model using confirmatory factor analysis that examines the relationships between the latent constructs and their indicator variables and (2) a structural model to test the hypothesized relationships between the latent mediating and outcome constructs and the intervention [62]. We will use the maximum likelihood method of parameter estimation with missing data. We will also conduct separate analyses to examine the effectiveness of intervention components in specific subgroups (eg, by gender).
- Aim 3: Qualitative analysis will be conducted in both Azerbaijani and English to explore barriers and facilitators to intervention participation and implementation. Bilingual investigators and 2 additional coders will independently review interview transcripts and conduct thematic analysis [63] and content analysis [64] using Dedoose [65]. Analyses will assess themes and concepts regarding participants’ and intervention facilitators’ perspectives on mental health needs, intervention engagement and acceptability, changes in family relationships and dynamics, and contextual and sociocultural factors influencing intervention delivery and outcomes.

In the data analysis, the primary outcomes include children’s mental health and socioemotional functioning. Secondary outcomes include cognitive and social processes associated with mental health, as well as functional outcomes related to academic performance and social functioning in the school setting. Specifically, primary mental health outcomes include child-reported depressive symptoms, posttraumatic stress symptoms, and self-esteem; caregiver- and teacher-reported ADHD symptoms and general socioemotional functioning such as internalizing and externalizing problems; and clinician-administered mental health outcomes among families randomized to the mental health services condition. Secondary outcomes include child cognitive functioning, assessed using WISC-V and CANTAB tasks; child-reported social support, particularly peer support; and teacher-reported grades and school behaviors. Mediating variables include caregiver-reported parenting practices, caregiver mental health, family functioning, coping strategies, social support, saving attitudes, and household economic well-being.

The main analyses focus on treatment effects at the 1-year and 2-year follow-ups, with the 1-year follow-up used to assess initial intervention effects and the 2-year follow-up used to assess the maintenance of those effects over time. Children from the same family will be accounted for using mixed-effects models, with random effects specified to adjust for the nonindependence of observations among children nested within the same family. Missing outcome data will be handled using maximum-likelihood estimation under a missing-at-random assumption, with sensitivity analyses using multiple imputation. To address the multiple testing issue, we will control the false discovery rate using the Benjamini-Hochberg procedure [66,67]. This approach was selected instead of Bonferroni correction because it limits false discoveries while avoiding the overly conservative loss of power associated with Bonferroni adjustment when multiple related hypotheses are tested.

### Ethical Considerations

Given the vulnerability of children with histories of institutional care and family separation, the study incorporates several safeguards to protect participants’ safety, privacy, and autonomy. Caregiver consent and child assent are obtained separately by trained Azerbaijani-speaking research staff to reduce coercion, and families are informed that participation is voluntary and that refusal or withdrawal at any time does not affect access to usual care or public mental health services. Adverse events are monitored, reported, and reviewed for systematic trends every 2 months. Research staff and intervention facilitators are trained mental health, psychology, or social work professionals, and procedures are in place to respond to emotional distress, including a General Distress Management Protocol and referral to the National Mental Health Centre when serious mental health concerns or risks of harm to self or others arise. Confidentiality and its limits are explained during consent and assent; although local law does not mandate research staff to report suspected child abuse or neglect, the study follows Child Safeguarding procedures for suspected child-safety incidents, including review by the local Project Director and Child Safeguarding team and referral to appropriate child protection authorities for serious cases. Institutional review boards at the University of Chicago (IRB20-1018) and Azerbaijan Medical University approved this study and must approve any future amendments to the protocol.

Compensation is modest and prorated to reimburse participants for their time, thereby reducing the possibility of coercion. Eligible participants received US $10 per person for each assessment and US $10 per family for each intervention session attended to compensate for their time and transportation costs. Compensation is provided after each completed visit or session, with partial compensation available for incomplete participation.

All identifiable information is anonymized with a unique study ID number, and only the PI and local project coordinator can access deidentified data. The PI and local project director monitored local data collection and management procedures regularly. The Data and Safety Monitoring Board, an independent committee responsible for ensuring participant safety and data integrity, conducted the monitoring of research processes in collaboration with mental health professionals, a biostatistician, and representatives from the Azerbaijan Medical University.

The investigative team has shared information about this trial via ClinicalTrials.gov, as per National Institutes of Health policy, and at key conferences. The team will develop a plan for dissemination at the national and regional levels (eg, brief reports). Once all data have been deidentified, cleaned, and validated, and the main findings have been published, the investigators expect to share data with the scientific community. To safeguard the protection of participants, datasets free of identifiers will be made available to individuals who directly contact the PI.

## Results

Recruitment was completed in 2023, and 2-year follow-up data collection was completed in 2025. The study enrolled 439 children and 305 of their caregiver participants from the 3 target sites, with 107/305 (35.1%) caregivers having more than 1 child participating in the study [68]. On average, children were 6.8 (SD 2.18) years old when they were placed in institutional care, with most placements occurring around the start of formal schooling, which institutional care facilities provide free of charge. The average duration of time in institutional care was close to 3 (mean 2.88, SD 2.39; range <1-12) years. Most participating caregivers are female 282/305 (92.5%) and predominantly mothers and grandmothers [68]. This sample exhibits economic instability, with 130/305 caregivers (42.6%) reporting that their family’s economic situation was bad or very bad [68,69].

Descriptive analyses of baseline data revealed substantial mental health needs among participating children [68]. At baseline, many children scored above clinical cutoffs for key mental health symptoms, including 221/425 (52%) for depressive symptoms, 156/429 (36.4%) for posttraumatic stress symptoms, and more than one-third for ADHD symptoms. Baseline analyses to date also suggest that economic instability and parental distress play important roles in child outcomes among this population [69]. Structural equation modeling analyses of baseline data identified an indirect pathway linking economic deprivation to child internalizing problems through parental distress and the family environment. Greater economic deprivation was associated with higher levels of parental distress (child-report model: *β*=.39, *P*<.001; parent-report model: *β*=.38, *P*<.001) which, in turn, was linked to a less supportive family environment (*β*=−.71, *P*<.001; *β*=−.69, *P*<.001, respectively). A more supportive family environment was associated with fewer child internalizing problems (*β*=−.29, *P*=.007; *β*=−.54, *P*<.001) [69], suggesting that parental distress and family functioning may represent important pathways through which economic hardship affects child mental health [69].

## Discussion

The study aims to test the hypotheses that one or more of the 3 interventions—family strengthening, mental health services, and economic empowerment—improve children’s mental health and socioemotional functioning at home and school at the 1-year and 2-year follow-ups. By using a full factorial design, the study seeks to identify the individual and combined effects of these interventions and determine the most optimal intervention package for promoting the psychosocial well-being of children transitioning from institutional to family-based care.

If the intervention components are found to be effective and feasible, the optimized intervention may help strengthen deinstitutionalization efforts by addressing care needs that are not fully covered by standard case management services. Current deinstitutionalization programs play an important role in transitioning children from institutional care to family settings; however, they may not fully address children’s mental health needs, caregiver stress, family relationships, or poverty-related factors that contributed to institutional placement [7, 21]. By testing family strengthening, trauma-focused mental health care, and economic empowerment, this study may provide evidence on whether these additional supports can improve child and family well-being during reintegration. Any future scale-up across Azerbaijan or similar regional contexts would require careful consideration of effectiveness, feasibility, cost, implementation capacity, and ethical safeguards.

This study addresses 2 Grand Challenges in Global Mental Health identified by the National Institute of Mental Health: (1) developing locally appropriate strategies to eliminate childhood abuse and enhance protection and (2) developing interventions to mitigate the long-term mental health impact of low childhood socioeconomic status. Additionally, by providing evidence-based insights, this research can guide the development of care models that prioritize family-based and community-centered alternatives in LMICs. The findings may help identify effective mental health interventions tailored to the unique needs of at-risk children, including those affected by poverty, social exclusion, and adverse childhood experiences. This research reinforces the global commitment to protect children’s well-being, ensuring that no child is left behind in accessing care and support.

The study has several unique strengths. These include the randomized factorial design, which allows the estimation of the individual and combined effects of individual interventions; the use of culturally adapted intervention materials; collaboration with local community partners; implementation in real-world community settings; longitudinal follow-up over 2 years; the assessment of outcomes across multiple domains and informants; as well as the integration of administrative records and digital, nonverbal, and predominantly language-independent cognitive paradigm tasks to supplement traditional self-report measures.

At the same time, several limitations should be considered. The study was conducted in a specific national and service-delivery context, which may affect generalizability to other settings. In addition, while the full factorial design is well suited to estimating the main effects of interventions, the study may have more limited power to detect smaller interaction effects or subgroup differences.

Future analyses will evaluate intervention effects at the 1-year and 2-year follow-ups, examine potential intervention mediators and moderators, and assess implementation-related factors such as acceptability, participation, fidelity, service provider experiences, and facilitators and barriers to intervention delivery. The findings will be disseminated through peer-reviewed publications, conference presentations, and reports or policy briefs for local and international stakeholders, including mental health, child welfare, and educational agencies, as well as community organizations and other relevant stakeholders in Azerbaijan and the broader region.

## Supplementary material

10.2196/93033 Multimedia Appendix 1 Caregiver consent and parental permission form.

10.2196/93033 Checklist 1 SPIRIT checklist.

10.2196/93033 Peer Review Report 1 Peer Review Report from ZMH1 - ERB-C (01) National Institute of Mental Health Special Emphasis Panel NIMH Social Drivers of Mental Illnesses in Low and Middle Income Countries RFA (National Institutes of Health, USA).

## Abbreviations

ADHD: attention-deficit/hyperactivity disorder
CANTAB: Cambridge Neuropsychological Test Automated Battery
CDA: Child Development Account
LMIC: low- and middle-income country
MOST: Multiphase Optimization Strategy
NMHC: National Mental Health Centre
PI: principal investigator
RA: research assistant
SPIRIT: Standard Protocol Items: Recommendations for Interventional Trials
WISC-V: Wechsler Intelligence Scale for Children, Fifth Edition
