# Supplementary material for: Optimizing Multicomponent Interventions to Improve Child Mental Health Following Reintegration From Institutions: Protocol for a Factorial Randomized Trial
Source: JMIR Res Protoc. 2026 Jul 16;15:e93033. doi: 10.2196/93033 (PMC13377521; doi:10.2196/93033)
Supplement: Multimedia Appendix 1 [file resprot-v15-e93033-s001.pdf]

The University of Chicago Crown Family School of Social Work, Policy, and Practice
National Mental Health Centre of Azerbaijan

CAREGIVER CONSENT and PARENTAL PERMISSION FORM

Protocol Number: IRB20-1018
Principal Investigators: Leyla Ismayilova, PhD, Fuad Ismayilov, MD
Title of IRB Protocol: Optimizing prevention approaches for children reintegrating from orphanages in Azerbaijan

CONTACT

Introduction

We invite you and one of your children aged 7-12 to participate in a research project studying how to help children who lived in orphanages and returned home to their families. The findings from this study will be used to inform the deinstitutionalization program in Azerbaijan. We are inviting you because your child used to live in an institution and returned home.

The purpose of this form is to give you information to help you decide whether you and your child would like to participate in the study or not. You will have a chance to ask questions about this research study, including your rights as a voluntary participant. When we have answered all your questions, you can decide if you want to be in the study or not. You will also be given a copy of this form for your records.

INFORMATION ON RESEARCH

Research Purpose

The purpose of this study is to test three different programs that focus on the difficulties that children from institutions and their families may face in their daily lives after reuniting. In this research project, we would like to understand if these new programs support families and help them deal with various problems when a child is returning home after living in an institution. The information you and your child provide will help us understand if any of these programs could assist these children to adjust better to the life outside of orphanages and to provide support to families who care for these children. This study is sponsored by the U.S. National Institute of Health and conducted together with the National Mental Health Centre, the SOS Children's Villages-Azerbaijan, and the University of Chicago.

Study Procedures

Interviews: We are asking you and your child to participate in three individual interviews (one per year) with a member of our research team, such as myself. During the individual interviews, we will ask you questions related to your daily life, your economic situation, education, work, your family situation and how you feel about yourself, your child and your family. We will also ask about your child's time in the institution (e.g., when your child was placed there, which type of institution), about your relationship and behavior with your child and any difficulties your child has at home, at

school or with friends. Your first interview can be conducted today, the second interview will be in about one year and the third interview in about two years from today.

Some of the questions we will ask you may be sensitive in nature such as those relating to violence and abuse.

Each interview with you will take about 60 minutes to complete and the interview with your child will take about 30-45 minutes. You and your child will have separate interviews. Your child will also be asked to do a few tasks on the computer or iPad (e.g., recall pictures, organize blocks similar to the picture). During the interview with your child, we will ask questions related to your child's daily life, schoolwork, his/her friends and your child's relationship with your family members or difficulties in these areas. We will be very careful to keep yours and your child's answers private. The information you and your child provide in these interviews will be used to understand if this program can help other families who have children in institutions.

This is not a test and there are no right or wrong answers to any of the questions. You or your child may refuse to participate or withdraw at any time. You and your child may refuse to answer or request to skip any question you do not feel comfortable answering. Your refusal will not affect the benefits to which you are otherwise entitled, such as government aid or services.

With your permission, we will contact your child's teacher at school to collect information about your child's school attendance, academic performance and behavior at school.

Programs: After the interview, your family will be selected in one of several groups. This process will be by chance like a lottery or flipping a coin. Depending on group, your family will be invited to participate in one of three programs: family group program, economic program or psychological help. It is possible that you will be selected to participate in more than one of these programs or none of these programs.

a. If you are selected to participate in the **family group program**, you will meet once a week for an hour together with a trainer. You will meet as a group with five other families with children who have also returned home from institutions. You can invite any family members you want to join the program with you. This could include any family members who live with you or who are involved in caring for your children (all your children, your spouse, your parents, your siblings or other family members who you trust and would like to participate in the program with you). The weekly group meetings will continue for 12 weeks. You will do different activities and talk with other group members and with your children about things that bother you about raising your child and how to support your child after returning home from an institution, how to discipline children and help them through difficult situations at home and at school. The program can also help you learn how to get along better as a family, resolve potential conflicts between children and parents, and how to better understand and communicate with your child after he/she spent some time living away from you. At the end of each session, we would also ask you to fill out a brief form (5-10) minutes rating whether you found each session interesting, informative, and useful. For participation in each family group session, your family will receive $10 (converted in manats) for each session (12 sessions in total). If your family attends all 12 sessions without missing, your family will receive up to $150, in total.

b. If you are selected to participate in the **psychological help program**, you and your child will meet with specialists (e.g., psychologist and psychiatrist) who would be able to meet with your child, assess any concerns your child has or you have about your child's behavior, and receive psychological assistance on how to improve or better manage these concerns. The sessions usually take place twice a week (30-45 min) and usually would last 2-3 months. If your child's difficulties persist, the services may be extended. Services will be provided the public mental health center and are free of charge. For attending sessions, your family will receive $10 (converted in manats) per session, for up to 12 sessions.

c. If you are selected to participate in the **economic program**, a savings bank account will be opened under your child's name (with the starting amount of $50 converted in manats). Your will attend eight sessions with other families from this program to learn how to save and set aside money for education, home or other important life decisions (e.g., starting a small business). Your family will receive $10 for attending each session. At the end of each session, we would also ask you to fill out a brief feedback form (5-10 minutes) rating whether you found each session interesting, informative, and useful. You will be encouraged to save and make monthly deposits that can be doubled (for up to $20 per month) in a separate account called Matched Account. In case of emergency or when necessary, you would be able to withdraw money that you deposited into the Child's Account. Money from the Matched Account that be taken only to pay for education, home or to invest in small business. After completing the sessions, your family will meet with your economic program facilitator once a month to review the bank statement from the child's and matching accounts, discuss if you are able to make contributions to the savings account and address any potential problems to achieving your financial plans. We will use information from your bank statements to understand how much families are able to save, on average.

If you participate in any of these programs, we would use information from your feedback forms together with your attendance and types of services to understand families' opinions about these programs.

RISKS

For this study, the primary risk is that you or your child may feel uncomfortable, upset or embarrassed when discussing sensitive or emotional topics such as separating from your child or circumstances that led to separation. Tell the interviewer at any time if you need to take a break or stop the interview. If you or your child do not wish to participate in the interview or choose to end your participation, you are allowed to do so at any time and for any reason.

You may also get upset if sensitive topics arise in the intervention sessions. The loss of confidentiality and disagreements between children and caregivers are possible during the family group sessions. All group participants will be reminded at the beginning of each intervention session that all information shared by other participants should remain confidential and should not be shared outside the group. By signing this form, participants agree not to talk about private information outside of the intervention session. Even with this agreement, someone may talk about your information outside of the intervention. It is also possible that issues may be raised by you or your child that might cause conflicts. Project staff members are trained to help families deal with conflict and help them resolve these issues. If you do not wish to participate in the study or choose

to end your participation, you are allowed to do so at any time, and for any reason. There may be other risks, which are not known at this time.

BENEFITS
You and your child may or may not benefit by participating in this study. You may potentially learn how to save money or how to understand and manage your child's behavior better. However, the information you provide may also help improve programs that could provide psychological and financial support to other families with children from orphanages.

ALTERNATIVE PROCEDURES
You may choose not to participate in this study. If you choose not to participate in the study, you may benefit from standard social and psychological services offered by the government.

CONFIDENTIALITY
All information that you provide is only for research purposes. Although every reasonable effort will be made to protect the confidentiality of your records, such protection cannot be absolutely guaranteed. We will do everything possible to maintain your confidentiality at all times. Your name or your child's name will not appear on materials related to this study; it will be replaced by a coded number. Only the research team will have access to information that identifies you to carry out this research study and for use in future studies [if applicable] without additional consent. Your identifying information will not be shared with others outside this research study. Participants may withdraw consent for research use of their data at any time without penalty or loss of benefits to which the participant is otherwise entitled. In this event, data will be withdrawn from any stored file, if possible, but data already distributed for research use will not be retrieved.

We will not share any of your answers with any of your friends, neighbors, family members, or public officials. In the same way, we will not share any information or answers we get from your child. The only exception to confidentiality would be the risk of immediate harm to yourself or others. If we think you or someone else in your home is at risk of immediate harm to self or others, we are required, by law, to report this to the authorities [by following the Child Safeguarding reporting procedures]. For example, if you tell us that you are going to hurt yourself or someone else such as your child or that someone is hurting or abusing you, in such circumstances we would have to notify the local authorities, such as the NMHC or Commission on Minors' Affairs and Protection of Their Rights. By signing this form, you also agree to not talk about private information outside of the group intervention sessions.

When disseminating information about this study, results will be provided as summary and your name will not be used in any report, conference, published articles or databases.

COMPENSATION
In appreciation of your time and to help compensate for your travel expenses, both you and your child will each receive $10 (converted into Azerbaijani manats) for each interview. If both you and your child participate in all three interviews over the two-year study period, you will receive a total of $90 together, equivalent in Azerbaijani manats.

ADDITIONAL COSTS
There will be no cost to you to participate in the study.

VOLUNTARY PARTICIPATION
Participation in this study is voluntary. You or your child can refuse to participate in the study at any time, for any reason and you would not be penalized in any way. You can also refuse to answer or skip any questions during the interviews, if they make you feel uncomfortable. If you and your child decide to participate in the study, but later change your mind, you may discontinue your participation at any time without any consequences of any kind.

ADDITIONAL INFORMATION
If you have any questions or concerns about the study, you may contact Dr. Narmin Guliyeva, the Project Director for this study in Baku at (050) 209-90-89 or you may call Dr. Leyla Ismayilova at University of Chicago in Chicago, USA at +1 (773) 834-0401. If you have questions about your rights as a research participant, you may contact the Ethics Committee at Azerbaijan Medical University (Dr. Rauf Beylarov, Chairman of the Ethics Committee of the AMU by phone (012) 597-37-23 or via email r.baylarov@ amu.edu.az)

Do you have any questions about this study?

PARTICIPANT'S STATEMENT OF CONSENT
I have read the above purpose of the study, and understand my role in participating in the research. I voluntary agree to take part in this study. I have had a chance to ask questions. If I have questions later about the research, I can ask the investigators listed above. I understand that I may refuse to participate or withdraw from participation at any time without any negative consequences. I have received a copy of this document for my records.

By checking one of the boxes below, I am indicating whether I voluntarily agree to participate in this study:

_____ Yes, I agree to participate in the study

_____ No, I do not agree to participate in the study

Signatures

Caregiver Participant

Print Name ______________________________ Date _______________

Signature ______________________________

Person Obtaining Consent

Print Name ______________________________ Date _______________

Signature ______________________________

PARTICIPANT'S STATEMENT OF PARENTAL PERMISSION
I understand my child's role in participating in this study. I give permission for my child to pursue his/her participation with this study. I have been given a chance to ask questions. I understand that my child can refuse to participate or can withdraw his/her participation at any time without any negative consequences. I certify that I freely give my consent for my child to participate with this study. I will receive a copy of this document for my records.

By checking one of the boxes below, I am indicating whether I voluntarily agree for my child to participate in this study:

_____ I agree for my child to participate in the study

_____ I do not agree for my child to participate in the study

Signatures

Child's Parent or Legal Guardian

Print Name ________________________________ Date ________________

Signature ________________________________

Child Participant

Print Name ________________________________

Person Obtaining Consent

Print Name ________________________________ Date ________________

Signature ________________________________
