# Supplementary material for: Optimizing Multicomponent Interventions to Improve Child Mental Health Following Reintegration From Institutions: Protocol for a Factorial Randomized Trial
Source: JMIR Res Protoc. 2026 Jul 16;15:e93033. doi: 10.2196/93033 (PMC13377521; doi:10.2196/93033)
Supplement: Peer Review Report 1 [file resprot-v15-e93033-s003.pdf]

SUMMARY STATEMENT
(Privileged Communication)

PROGRAM CONTACT:
Holly Campbell-Rosen
240-627-3316
holly.campbell-rosen@nih.gov

Release Date: 07/26/2023
Revised Date:

Application Number: 1R01MH135095-01

Principal Investigator

ISMAYILOVA, LEYLA

Applicant Organization: UNIVERSITY OF CHICAGO

Review Group: ZMH1 ERB-C (01)
National Institute of Mental Health Special Emphasis Panel
NIMH Social Drivers of Mental Illnesses in Low and Middle Income Countries RFA

Meeting Date: 06/22/2023
Council: OCT 2023
Requested Start: 12/01/2023
Opportunity Number: RFA-MH-23-310
PCC: 9G-GMH4

Project Title: Optimizing Multi-level Interventions to Improve Child Mental Health in Azerbaijan

SRG Action: Impact Score:18
Next Steps: Visit https://grants.nih.gov/grants/next_steps.htm
Human Subjects: 30-Human subjects involved - Certified, no SRG concerns
Animal Subjects: 10-No live vertebrate animals involved for competing appl.
Gender: 1A-Both genders, scientifically acceptable
Minority: 5A-Only foreign subjects, scientifically acceptable
Age: 2A-Only Children, scientifically acceptable

Project Year	Direct Costs Requested	Estimated Total Cost
1	499,999	676,587
2	499,998	676,586
3	500,000	676,588
4	499,999	676,587
5	499,998	676,586
TOTAL	2,499,994	3,382,934

1R01MH135095-01 ISMAYILOVA, LEYLA

ADMINISTRATIVE NOTE

RESUME AND SUMMARY OF DISCUSSION:
This is a new application received in response to the Social Drivers of Mental Illnesses in Low- & Middle-Income Countries (LMICs): Mechanisms and Pathways of Interventions for Youth (R01) RFA-MH-23-310. This application proposes to improve clinical mental health, cognitive and social outcomes among children (ages 7–14 years) from low-income families in Azerbaijan, by adapting and testing three evidence-based intervention approaches: (a) a family-strengthening intervention; b) trauma-focused mental health care for parents; and c) economic empowerment in the form of Child Savings Accounts. The interventions would be tested among 600 child-caregiver dyads using a multiphase optimization strategy (MOST) design. This application possesses a variety of notable strengths. In particular, the application includes an exceptional investigative team with a successful history of collaboration, and an equally strong environment in which to conduct the proposed study. The investigative team includes a well-established and well-connected local research team in Azerbaijan. The application also includes an outstanding approach that contributes to the feasibility of scaling up interventions and is considered a strength. Another strength is the study's innovation, including the proposed use of a multiphase optimization strategy to test three interventions, and the automated computerized assessments, particularly in LMICs. Together with these strengths, a few minor weaknesses exist. The application might have provided additional evidence that recruitment goals are attainable; however, the investigative team successfully recruited participants in the local area in previous studies. There are also some questions related to potential overlap between the proposed study and prior studies conducted by the investigative team, and several aspects of the application might have been better described (e.g., demographic characteristics of intended participants, how demographic differences across sites would be addressed, Aim 2 and 3 analyses, study milestones). Overall, these weaknesses were considered minor in what is otherwise an outstanding application presenting a highly innovative approach from a well-qualified team.

DESCRIPTION (provided by applicant):
Adversity, economic deprivation, poverty-induced stress, prolonged exposure to trauma and parental distress increases the risk of child maltreatment and may severely undermine the mental health functioning of children. While there are efficacious interventions targeting individual, family, and structural-level factors associated with children's emotional and behavioral well-being, it is unknown how these interventions interact and complement each other and whether their effects can be synergistic (i.e., if the interventions' combined effects are greater than the sum of the individual effects). To improve mental health outcomes among children aged 7–14 from low-income families in Azerbaijan, the proposed study will refine and test three evidence-based intervention approaches: (a) family-strengthening intervention; b) trauma-focused mental health care for parents; and c) economic empowerment in the form of Child Savings Accounts. These interventions have been adapted to the context of Azerbaijan. The adapted interventions will be tested with 600 child-caregiver dyads in a trial using the Multiphase Optimization Strategy (MOST) to compare different intervention components and identify the most optimal combination. Given the limited human and financial resources in the LMICs, it will be important to identify whether each of these interventions is necessary and/or sufficient for improving the mental health of children. The study will test the effects of each

intervention component on children's clinical mental health outcomes (symptoms of depression, anxiety; disruptive behaviors; post-traumatic symptoms and on cognitive and social processes associated with these outcomes (e.g., attention, inhibitory control, working memory, emotion recognition bias). The study will also examine the mediating pathways associated with each intervention component. If successful, the study results could inform the National Mental Health Care reforms implemented by the Ministry of Health, WHO, UNICEF and other organizations in Azerbaijan and other post-Soviet countries. The proposed study addresses the NIMH's Strategic Plan Objective 3.2.A by tailoring existing interventions to optimize outcomes, and targets two top challenges within the NIMH Grand Challenges in Global Mental Health Goal B (Advance prevention and implementation of early interventions): 1) to develop locally appropriate strategies to eliminate childhood abuse and enhance child protection and 2) to develop interventions to reduce the long-term negative impact of low childhood socioeconomic status on mental health. Through training, mentoring and collaborative research, the project also aims to strengthen local research expertise in: 1) the core components and mechanisms of change of psychosocial mental health interventions for children and families living in conditions of chronic adversity; 2) the adaptation of multilevel evidence-based interventions and measurement tools to the new socio-cultural context of a post-Soviet country; and 3) methodological and statistical techniques for evaluating complex interventions with multiple components.

PUBLIC HEALTH RELEVANCE:
Adverse conditions such as economic deprivation, complex trauma, and chronic parental stress increase the risk that children may be mistreated and may severely undermine their mental health functioning. Many post-Soviet countries struggle with a legacy of a medicalized approach to mental health care that does not address the social and structural factors that contribute to poor mental health. Advancing the mental health of children in post-Soviet Eurasia requires research and capacity building activities that will strengthen collaborative international and regional community–academic partnerships and build expertise to adapt, integrate, and compare multi-level psychosocial interventions that address the underlying family-level and structural risks and protective factors related to child mental health.

CRITIQUE 1

Significance: 1
Investigator(s): 1
Innovation: 3
Approach: 2
Environment: 1

Overall Impact:
This is an excellent application that has potential for significant impact to improve youth mental health in Azerbaijan by shifting from a medical model of clinical interventions to a preventive model targeting social factors that influence mental health. The aims are clear and easy to follow. The application proposes to use the MOST strategy (Multiphase Optimization Strategy) to: 1) compare effects of 3 interventions (family strengthening, economic empowerment, trauma-focused mental health care for parents) on mental health, cognition and social processes, and functioning (e.g., school performance) among youth 7-14; 2) examine

mediators and moderators of mental health; and 3) explore barriers and facilitators to implementation of each intervention. The design, investigative team, and environment are all strong. The opportunity to target the combination of interventions that most effectively prevent poor mental health among youth in post-Soviet countries is compelling. The multi-level approach incorporates qualitative information to adapt existing interventions and anticipates scale-up by exploring implementation barriers/facilitators and costs, as well a post-study conference to disseminate findings to similar countries. There are a couple of minor points: 1) there might have been better clarity on the degree of overlap with the existing R01 (how novel is the MOST strategy if the same team has used it with the same interventions before?); and 2) analyses for Aim 3 do not appear to be presented in the Research Strategy.

1. Significance:
Strengths

- The proposal to shift from a medicalized intervention model to a preventive model that will mitigate the emergence and evolution of longer-term sequalae is a strength.
- The focus on multiple intervention paths, including economic drivers and the impact of parent mental health, has potential to elucidate the optimal combination of intervention points for broader scale-up.
- The study setting offers opportunity for significant impact: its location (Eurasia) and geopolitical climate (post-Soviet country with current migration and refugee crises) are not often part of global mental health studies.

Weaknesses

- Azerbaijan is an upper-middle income country. While this does fit the criteria – and does make it unique –findings appear likely to be less generalizable to lower resource settings.

2. Investigator(s):
Strengths

- The investigative team is excellent: The PI is a clinical social worker and Associate Professor at University of Chicago with experience leading an R01 and other studies. Notably, the PI grew up in Azerbaijan, speaks the local language, and is familiar with the culture, religion, and other important social aspects.
- Co-Is at the University of Chicago (U of Chicago) are established researchers in psychology and quantitative psychology. One of the Co-Is works with the developer of the MOST strategy that is being employed.
- Azerbaijan-based Co-Is are well-suited to execute the data collection and research, including partners at the National Mental Health Centre who are experienced clinicians as well as experienced with NIH-supported research with the PI.
- Co-I at SOS Children's Village (Soltanov) is Director of SOS Villages in Azerbaijan, where participants will be recruited, and has experience conducting research with the PI.
- US-based co-Investigators reflect other research-oriented US-based institutions and expertise that will benefit the application – Dr. Ssewamala (Washington University) is a senior consultant with experience adapting interventions and economic strengthening. Dr. Munir (Boston Children's) is a child psychiatrist originally from Turkey, where language and culture are similar to that of Azerbaijan.
- This is a well-established U Chicago and Azerbaijani research team with history of preliminary studies motivating the current proposal.

Weaknesses

- None noted.

3. Innovation:

Strengths

- The study adapts and integrates 3 multi-level interventions to assess what combination nets the optimal outcomes in a post-Soviet country.
- The study incorporates a computerized task system to assess memory, attention, response inhibition and emotion recognition – which supplements self-report data with a more objective measure.

Weaknesses

- It's not entirely clear how much this overlaps with the existing R01 – which at different points in the application seems to be described as providing the same interventions and employing the MOST strategy for integration optimization.

4. Approach:

Strengths

- Broadly, the approach is very well-organized, easy to read, logical, and coherently presented.
- Preliminary studies - including qualitative exploration and quantitative intervention have provided a substantial evidence base to motivate the current research and have solidified the U Chicago and Azerbaijani researchers and local partners in ongoing collaborations.
- Anticipating broader scale up by building capacity among National Mental Health Centre (NMHC) partners across the country is a strength.
- Establishing a local community group of numerous types of stakeholders to inform adaptations is a plus.
- Computerized tasks to map self-reported cognitive functioning is a unique added feature that will offer benefits to the measurement knowledge base beyond the intervention's optimization.
- The study design is strong, includes multi-level aspects, and will enable assessing which aspects of intervention lead to the best outcomes.
- Adapting and combining existing evidence-based interventions with which the study team has experience makes an ambitious application feasible and will elucidate optimized interventions.
- The study includes an RDoC-like approach (early intervention to affect longer-term outcomes, computerized testing of cognition, caregiver health and poverty intervention targets) and is well-aligned with overall RDoC framework.
- The Aim 1 analytical approach is appropriate, though could use more detail on fixed/random effects specification (also see question in weaknesses about sites). In addition to p-values, the application might have reported the actual effect estimate and precision (CI).
- A post-study conference to share findings with other post-Soviet nearby countries where the optimized intervention may be generalizable/transportable is a plus.
- Follow-up time points to 2 years is a strength.
- Including QA/QC measures defined at the outset is a strength. However, rather than examine a 20% random sample after data collection is done, there might have been

greater consideration provided to assessing initial data for a fixed number of records to correct any issues that can be caught early.

Weaknesses

- The family strengthening intervention allows “all interested family members – parents, grandparents, aunts or uncles” to participate (which is a real strength). However, it is not fully clear whether ‘family’ only is defined by biology or whether close friends/neighbors who may be even more essential to the parent (especially a single mother) might be included. It seems that for incorporating the social, multi-family environment this would be important.
- Recruitment: It might have been helpful to specify how often families request services from the 3 National Mental Health Center (NMHC) sites proposed for study to offer assurance that 600 families can be enrolled in the recruitment period.
- Sites: It is not fully clear whether important social differences exist between the 3 study sites where families will be recruited and how these differences might affect adaptations to the intervention by site, and how this would be accounted for in analyses. Related, it is not entirely clear how the post-intervention qualitative interviews will ensure representativeness from different conditions at each site.
- Aim 2 analyses might have been better specified. How mediators are conceptualized on the causal path does not appear to be described with sufficient clarity. Traditionally, they would be between exposure and outcome (in this case: intervention—mediator—outcomes). Given that, it is not fully clear what measurement time points are conceptualized for the proposed modeling.
- Aim 3: Even though exploratory, it would appear important for there to be some offering of what will be done with Aim 3 primary data in the Research Strategy. It would also appear important for the cost analysis to be in the Research Strategy. Both appear to be in human subjects, but seem to deserve space in the Research Strategy.

5. Environment:

Strengths

- There is a strong institutional environment at U Chicago for conducting large-scale research, including data systems, security, IRB, etc.
- The National Mental Health Center (NMHC) in Azerbaijan is well-connected with the ministry of health (in which it is situated) as well as universities; the NMHC has participated in NIH-supported research and demonstrated capacity for the full range of implementation that entails.
- Similarly, SOS Children’s Villages has participated in research. More importantly, they provide the setting for conducting the research and are well-poised to facilitate recruitment and data collection while employing appropriate human subjects’ protocols.

Weaknesses

- None noted.

Study Timeline:

Strengths

- The timeline would normally seem overly ambitious, but it seems the research team has implemented these interventions previously (and done so together), which will make it much more feasible.

Weaknesses

- There appears to be no explicit comment on the study timeline wherein the application offers an assessment of whey the believe it is feasible and strategies for optimizing existing relationships, etc.

Protections for Human Subjects:
Acceptable Risks and/or Adequate Protections

- All human subjects sections are reasonable; consent, discomfort with questions, handling adverse events, etc. are covered.

Data and Safety Monitoring Plan (Applicable for Clinical Trials Only):
Acceptable.

- The DSMB is well-described and includes local and US partners. It might have been ideal to specify the frequency (or relevant junctures) of DSMB meetings.

Inclusion of Women, Minorities, and Individuals Across the Lifespan:

- Sex/Gender: Distribution justified scientifically.
- Race/Ethnicity: Distribution justified scientifically.
- For NIH-Defined Phase III trials, Plans for valid design and analysis: Not applicable.
- Inclusion/Exclusion Based on Age: Distribution justified scientifically.
- The population is White or more than one race, and the numbers reflect that. The planned enrollment report indicates more males, though the justification indicates more females are expected because there are more single mothers. Presumably, the male/female numbers were flipped and it’s a typographical error. The justification makes sense.

Vertebrate Animals:
Not Applicable (No Vertebrate Animals).

Biohazards:
Not Applicable (No Biohazards).

Applications from Foreign Organizations:
Justified.

- US-based PI with foreign organization co-Investigators. The research will advance potentially scalable and impactful interventions that target social drivers of mental health among post-Soviet populations.

Resource Sharing Plans:
Acceptable.

- There are no model organisms. Resource sharing of tools seems appropriate.

Budget and Period of Support:
Recommend as Requested

CRITIQUE 2

Significance: 2
Investigator(s): 1

Innovation: 3
Approach: 3
Environment: 1

Overall Impact:

This is a new application to examine multi-level interventions, including family-strengthening, trauma-focused mental health care for parents, and economic empowerment in the form of child savings accounts on child mental health outcomes of those aged 7-14 years of age in Azerbaijan. The study will test these interventions on differential outcomes with 600 child-parent dyads with elevated mental health problems in a trial using the Multiphase Optimizing Strategy (MOST). The study design recognizes the mental health service deficits and challenges while effectively building upon local strengths, including collaboration with the National Mental Health Center and the SOS Children's Village and their experienced collaborators. The assembled team, drawn from multiple institutions is appropriate, as the proposed investigation is enhanced by a history of successful collaborations among many of the team members and a shared deep appreciation of the child mental health needs and challenges in Azerbaijan. The Principal Investigator has the experience, expertise and required proven collaborations to successfully carry out the proposed project.

1. Significance:
Strengths
- This study proposes to make an important contribution to understanding the differential contribution of three common interventions, within one trial: family strengthening, economic empowerment, and trauma-focused care of parents.
- Plans to examine direct and indirect pathways of each of the three planned interventions is an important, integral part of the investigation.

Weaknesses
- None noted.

2. Investigator(s):
Strengths
- The PI has an outstanding record of research relevant to this proposed investigation.
- The research team, including investigators in the United States and in Azerbaijan are highly experienced researchers.
- Each institution/collaboration site has a proven record relevant to the investigation.
- The investigators have previously successfully collaborated.

Weaknesses
- None noted.

3. Innovation:
Strengths
- Testing multi-level interventions in Azerbaijan, a country with a previous, overly medicalized approach to mental health care, has significant, positive implications for this country as well as other post-Soviet countries.
- The offer to host a collaborative meeting of all awardees under this NIMH mechanism has great potential to immediately transfer knowledge across low- and middle-income countries (LMICs), within the immediate area and elsewhere.

Weaknesses
- None noted.

4. Approach:
Strengths
- Use of the Multiphase Optimization Strategy (MOST) is appropriate and useful for the interventions proposed.
- The study design is appropriate to the proposed objectives.
- Inclusion of facilitating factors and barriers based on qualitative interviews with participants and service providers adds to the strength of design.
- All proposed measures are valid and reliable; most are culturally adapted and others will be adapted to Azerbaijan.

Weaknesses
- It is not always fully clear where the previous research ends, and this proposed study begins.
- The proposed use of Qualtrics (rather than Recap which is also being used) could be problematic given that the data collected with this method is retained by Qualtrics, and therefore, appears vulnerable to loss of privacy.
- It’s not entirely clear how information about multiple children from a single family will be dealt with.
- There does not appear to be sufficient information regarding benchmarks to which study progress can be objectively assessed.

5. Environment:
Strengths
- The National Mental Health Centre (NMHC), operating under the Ministry of Health and Directorship of Fuad Ismayilov in Baku, Azerbaijan affords strong local support and proven leadership.
- The relevant research record of Narmin Guliyeva, also at NMHC demonstrates experience with the proposed research methodologies.
- The University of Chicago, site of the PI, is renowned for its scientific rigor, providing a sound and supportive environment for the proposed research.

Weaknesses
- None noted.

Study Timeline:
Strengths
- All requirements, including start up, implementation, recruitment, timelines, etc., of a good trial are met in this application.

Weaknesses
- None noted.

Protections for Human Subjects:
Acceptable Risks and/or Adequate Protections
- No comments provided.

Data and Safety Monitoring Plan (Applicable for Clinical Trials Only):
Acceptable.
- No comments provided.

Inclusion of Women, Minorities, and Individuals Across the Lifespan:
- Sex/Gender: Distribution justified scientifically
- Race/Ethnicity: Distribution justified scientifically
- For NIH-Defined Phase III trials, Plans for valid design and analysis: Not applicable.
- Inclusion/Exclusion Based on Age: Distribution justified scientifically.
- No comments provided.

Vertebrate Animals:
Not Applicable (No Vertebrate Animals).

Biohazards:
Not Applicable (No Biohazards).

Applications from Foreign Organizations:
Justified.
- Azerbaijan provides an excellent setting to carry out the proposed investigation, which should yield important contributions to understanding universal factors related to child/family mental health, particularly among those living in low-income areas.

Resource Sharing Plans:
Acceptable.

Budget and Period of Support:
Recommend as Requested

CRITIQUE 3

Significance: 1
Investigator(s): 1
Innovation: 1
Approach: 2
Environment: 1

Overall Impact:
Drawing on multiple evidence-based interventions with a view toward identifying the best way to combine approaches aiming to address the well-being of children and families beset by economic deprivation, complex trauma, and chronic stress, and building on an ongoing similarly-structured study of children in orphanages, this application outlines plans for a multiphase optimization strategy trial (MOST) in Azerbaijan to investigate the combined effects of a family-strengthening counseling intervention, a trauma-focused mental-health-care intervention, and economic empowerment through the use of child savings accounts. The principal investigator, Dr. Ismayilova, who is based at the University of Chicago, has extensive experience studying emotional well-being in Central Asia, and the research team includes world-class researchers at the University of Chicago as well as a network in Azerbaijan featuring affiliates of the National Mental Health Centre, the Department of Psychiatry at Azerbaijan Medical University, and the leadership of the non-profit organization SOS

Children's Villages. In addition to the innovation of the MOST study design, the project includes plans for collection of neuropsychological measurements allowing for investigation of mechanisms of interventions through emotion regulation, response inhibition, and other potential mediators. In addition, pilot data establishes both extensive mental-health need and evidence suggestive of meaningful intervention effects, and in addition to the study design being well-conceived, the application is thorough in its description of research and data-analysis plans. Overall, the project offers great potential to generate novel information that is apt to generalize to other post-Soviet countries, and along with highlighting its alignment with NIMH strategic-planning goals, the application convincingly highlights its potential to strengthen local research expertise.

1. Significance:
Strengths
- Political, economic, and refugee crises in Azerbaijan have induced conditions making it appealing and important to study the impact of social stressors on mental health.
- Pilot data show substantial rates of depression and trauma exposure among children in Azerbaijan.
- The application convincingly argues that clinical interventions alone are apt to be insufficient to address unmet need, and similarly that economic interventions alone are apt to be insufficient to address unmet need.
- The mix of economic deprivation, complex trauma, and chronic stress seen in Azerbaijan is emblematic of conditions in other post-Soviet countries, where few global mental-health studies have been conducted, suggesting that findings could readily and helpfully generalize to multiple other similar settings.

Weaknesses
- None noted.

2. Investigator(s):
Strengths
- PI Dr. Ismayilova has extensive experience studying emotional well-being internationally, including as PI of ongoing NIH-supported research on the mental health of children in orphanages in Azerbaijan, and her fluency in Azerbaijani and Russian language and familiarity with the culture can not only be expected to enhance the prospects of success for the project, but also to communicating effectively to build capacity in other post-Soviet countries.
- Dr. Deborah Gorman-Smith has key expertise relevant to project interventions, and her role as Dean of the Crown Family School of Social Work, Policy and Practice at the University of Chicago underscores institutional support for the project.
- Dr. Don Hedeker brings world-class biostatistical expertise to the project, including extensive familiarity with MOST designs, and also has a record of working with Dr. Ismayilova on her ongoing research in Azerbaijan.
- The project features outstanding expertise at Azerbaijan Medical University, notably including Dr. Salayev, Dr. Mammadzada, Dr. Guliyeva, and Dr. Faud Ismayilov (who also is Director of the National Mental Health Centre).
- The project also features relevant support in Azerbaijan from Zakir Soltanov, National Director of partnering agency SOS Children's Villages-Azerbaijan.

- Subcontracts to Azerbaijan Medical University and SOS Children's Villages include funding for numerous support staff who can be expected to contribute to the success of the project.

Weaknesses
- None noted.

3. Innovation:
Strengths
- The use of a MOST study design is a highly innovative strategy for investigating the impact of evidence-based interventions in combination with one another.
- Investigation of intervention effects in the post-Soviet environment of Azerbaijan, which has a history of a medicalized approach to mental health care, and which features an environment with numerous social stressors, is apt to provide novel yet generalizable insights applicable to other post-Soviet settings.
- The use of the Cambridge Neuropsychological Test Automated Battery offers innovative opportunities to investigate mechanisms of intervention effects through measurement of working memory, attention, response inhibition, and emotion regulation.

Weaknesses
- None noted.

4. Approach:
Strengths
- The proposed study design, building on the MOST design framework, provides an excellent foundation for the proposed research.
- The application provides an excellent overview and helpful relevant detail about each intervention.
- Pilot data (page 173) provides convincing evidence of the feasibility of the proposed research as well as effect size information, underscoring the likelihood of useful insights emerging from the proposed project.
- The inclusion of mental health clinical outcomes, cognitive outcomes, and functional outcomes can be expected to provide a comprehensive assessment of the impacts of interventions.
- Power calculations, which are anchored to the intervention with the smallest effect size, provide a solid foundation for the proposed project.
- The project includes appropriate data analysis plans.
- Qualitative interviews with participants and service providers can be expected to yield insights into facilitating factors and barriers to successful intervention.
- The inclusion of a Community Collaborative Board with experts in child mental health and social services can be expected to enhance the prospects of success for the project.
- The consideration of intervention fidelity through quality-assurance assessments can be expected to enhance the prospects of success for the project.

Weaknesses
- It is not entirely clear that the randomization to intervention combinations will be done in a way that can be expected to balance characteristics, such as the gender mix or age mix of participating children.
- The application does not adequately discuss plans to address multiple-testing considerations.

5. Environment:
Strengths
- The environment at the University of Chicago is clearly conducive to the success of the kind of research proposed.
- The environments of partnering institutions appear extremely well-suited to supporting the proposed project plans.

Weaknesses
- None noted.

Study Timeline:
Strengths
- The study timeline provides substantial detail pointing to the feasibility of the proposed activities.

Weaknesses
- None noted.

Protections for Human Subjects:
Acceptable Risks and/or Adequate Protections
- The project includes plans for informed consent to be administered by project staff rather than clinical staff, and for children to be consented separately from parents to avoid possible coercion to participate.

Data and Safety Monitoring Plan (Applicable for Clinical Trials Only):
Acceptable.
- The project includes plans for a data safety monitoring board and includes adequate monitoring of risks.

Inclusion of Women, Minorities, and Individuals Across the Lifespan:
- Sex/Gender: Distribution justified scientifically.
- Race/Ethnicity: Distribution justified scientifically.
- For NIH-Defined Phase III trials, Plans for valid design and analysis: Not applicable.
- Inclusion/Exclusion Based on Age: Distribution justified scientifically.
- The focus on children is motivated by the applied context, and the project will also include parents as participants.

Vertebrate Animals:
Not Applicable (No Vertebrate Animals).

Biohazards:
Not Applicable (No Biohazards).

Applications from Foreign Organizations:
Justified.
- Foreign justification underscores the merit of pursuing the proposed research in a post-Soviet country like Azerbaijan.

Resource Sharing Plans:
Acceptable.

Budget and Period of Support:
Recommend as Requested

THE FOLLOWING SECTIONS WERE PREPARED BY THE SCIENTIFIC REVIEW OFFICER TO SUMMARIZE THE OUTCOME OF DISCUSSIONS OF THE REVIEW COMMITTEE, OR REVIEWERS' WRITTEN CRITIQUES, ON THE FOLLOWING ISSUES:

ADMINISTRATIVE NOTE: Internet link/barcode figures or uniform resource locators (URL) have been inappropriately included in the application. The inclusion of URLs is not allowed as indicated in the PHS SF424 (R&R) Application Guide. As noted in NOT-OD-20-174, applications that include unallowable hyperlinks may be withdrawn from review and funding consideration.

PROTECTION OF HUMAN SUBJECTS: ACCEPTABLE.

INCLUSION OF WOMEN PLAN: ACCEPTABLE.

INCLUSION OF MINORITIES PLAN: ACCEPTABLE.

INCLUSION ACROSS THE LIFESPAN: ACCEPTABLE.

COMMITTEE BUDGET RECOMMENDATIONS: The budget was recommended as requested.

Footnotes for 1R01MH135095-01; PI Name: Ismayilova, Leyla

NIH has modified its policy regarding the receipt of resubmissions (amended applications).See Guide Notice NOT-OD-18-197 at https://grants.nih.gov/grants/guide/notice-files/NOT-OD-18-197.html. The impact/priority score is calculated after discussion of an application by averaging the overall scores (1-9) given by all voting reviewers on the committee and multiplying by 10. The criterion scores are submitted prior to the meeting by the individual reviewers assigned to an application, and are not discussed specifically at the review meeting or calculated into the overall impact score. Some applications also receive a percentile ranking. For details on the review process, see http://grants.nih.gov/grants/peer_review_process.htm#scoring.

MEETING ROSTER

National Institute of Mental Health Special Emphasis Panel
NATIONAL INSTITUTE OF MENTAL HEALTH
NIMH Social Drivers of Mental Illnesses in Low and Middle Income Countries RFA

ZMH1 ERB-C (01)
06/22/2023

Notice of NIH Policy to All Applicants: Meeting rosters are provided for information purposes only. Applicant investigators and institutional officials must not communicate directly with study section members about an application before or after the review. Failure to observe this policy will create a serious breach of integrity in the peer review process, and may lead to actions outlined in NOT-OD-22-044 at https://grants.nih.gov/grants/guide/notice-files/NOT-OD-22-044.html, including removal of the application from immediate review.

CHAIRPERSON(S)

SHERR, KENNETH, PHD, MPH
PROFESSOR
DEPARTMENT OF GLOBAL HEALTH
SCHOOLS OF MEDICINE AND PUBLIC HEALTH
UNIVERSITY OF WASHINGTON
SEATTLE, WA 98105

MEMBERS

BELIN, THOMAS, PHD
PROFESSOR
DEPARTMENT OF BIOSTATISTICS
FIELDING SCHOOL OF PUBLIC HEALTH
UNIVERSITY OF CALIFORNIA, LOS ANGELES
LOS ANGELES, CA 90095

CALLANDS, TAMORA A, PHD
ASSISTANT PROFESSOR
COLLEGE OF PUBLIC HEALTH
THE UNIVERSITY OF GEORGIA
ATHENS, GA 30606

CREA, THOMAS M., PHD
GLOBAL PROGRAMS
SCHOOL OF SOCIAL WORK
BOSTON COLLEGE
CHESTNUT HILL, MA 02467

GRAY, CHRISTINE L, MPH, PHD
ASSISTANT RESEARCH PROFESSOR
CENTER FOR HEALTH POLICY AND INEQUALITIES RESEARCH
DUKE UNIVERSITY
DURHAM, NC 27710

HOVEN, CHRISTINA W., MPH
PROFESSOR
DEPARTMENT OF CHILD PSYCHIATRY
NEW YORK STATE PSYCHIATRIC INSTITUTE
COLUMBIA UNIVERSITY
NEW YORK, NY 10032

IHEANACHO, THEDDEUS
ASSOCIATE PROFESSOR
YALE UNIVERSITY
YALE SCHOOL OF PUBLIC HEALTH
ORANGE, CT 06477

KARASZ, ALISON, PHD
PROFESSOR
DEPARTMENT OF FAMILY MEDICINE AND COMMUNITY HEALTH
UNIVERSITY OF MASSACHUSETTS CHAN MEDICAL SCHOOL
WORCESTER, MA 01655

MCBAIN, RYAN K, MPH
PROFESSOR OF POLICY ANALYSIS
RAND CORPORATION
WASHINGTON OFFICE
ARLINGTON, VA 22202

NADEEM, ERUM, PHD
ASSOCIATE PROFESSOR
GRADUATE SCHOOL OF APPLIED & PROFESSIONAL PSYCHOLO
RUTGERS UNIVERSITY
PISCATAWAY, NJ 08854

PEREZ-EDGAR, KORALY E, PHD
PROFESSOR
DEPARTMENT OF PSYCHOLOGY
COLLEGE OF THE LIBERAL ARTS
PENNSYLVANIA STATE UNIVERSITY
UNIVERSITY PARK, PA 16801

SENSOY BAHAR, OZGE, PHD
RESEARCH ASSISTANT PROFESSOR
GW BROWN SCHOOL OF SOCIAL WORK
WASHINGTON UNIVERSITY IN ST. LOUIS
ST. LOUIS, MO 63130

SIDDIQUE, JUNED
PROFESSOR
DEPARTMENTS OF PREVENTIVE MEDICINE (BIOSTATISTICS)
AND PSYCHIATRY AND BEHAVIORAL SCIENCES
NORTHWESTERN UNIVERSITY
FEINBURG SCHOOL OF MEDICINE
CHICAGO, IL 60611

STORY, WILLIAM THOMAS, MPH, PHD
ASSISTANT PROFESSOR
COLLEGE OF PUBLIC HEALTH
UNIVERSITY OF IOWA
IOWA CITY, IA 52242

WOLK, COURTNEY LYNN BENJAMIN, PHD
ASSISTANT PROFESSOR
DEPARTMENT OF PSYCHIATRY
PERELMAN SCHOOL OF MEDICINE
UNIVERSITY OF PENNSYLVANIA
PHILADELPHIA, PA 19104

SCIENTIFIC REVIEW OFFICER

ORTIZ, CLAUDIO DARIO
SCIENTIFIC REVIEW OFFICER
REVIEW BRANCH
DIVISION OF EXTRAMURAL ACTIVITIES
NATIONAL INSTITUTE OF MENTAL HEALTH
NATIONAL INSTITUTES OF HEALTH
BETHESDA, MD 20892

EXTRAMURAL SUPPORT ASSISTANT

WORTHAM, LYCURTIS
PROGRAM SPECIALIST
DIVISION OF EXTRAMURAL ACTIVITIES
NATIONAL INSTITUTE OF MENTAL HEALTH
NATIONAL INSTITUTES OF HEALTH
BETHESDA, MD 20892

Consultants are required to absent themselves from the room during the review of any application if their presence would constitute or appear to constitute a conflict of interest.
